# Treatment with fibroblast growth factor 19 increases skeletal muscle fiber size, ameliorates metabolic perturbations and hepatic inflammation in 5/6 nephrectomized mice

**DOI:** 10.1038/s41598-023-31874-4

**Published:** 2023-04-04

**Authors:** Berengère Benoit, Alice Beau, Émilie Bres, Stéphanie Chanon, Claudie Pinteur, Aurélie Vieille-Marchiset, Audrey Jalabert, Hao Zhang, Priyanka Garg, Maura Strigini, Laurence Vico, Jérôme Ruzzin, Hubert Vidal, Laetitia Koppe

**Affiliations:** 1 CarMeN Laboratory, INSERM, INRAE, Claude Bernard Lyon 1 University, Pierre Bénite, France; 2 Department of Nephrology and Nutrition, Hospices Civils de Lyon, Centre Hospitalier Lyon-Sud, Chemin du Grand Revoyet, 69495 Pierre Bénite, France; 3 INSERM U1059, Sainbiose, Jean Monnet University, Saint-Etienne, France; 4 Department of Molecular Medicine, Institute of Basic Medical Sciences, Faculty of Medicine, University of Oslo, Oslo, Norway

**Keywords:** Kidney diseases, Musculoskeletal system

## Abstract

Chronic kidney disease (CKD) is associated with osteosarcopenia, and because a physical decline in patients correlates with an increased risk of morbidity, an improvement of the musculoskeletal system is expected to improve morbi-mortality. We recently uncovered that the intestinal hormone Fibroblast Growth Factor 19 (FGF19) is able to promote skeletal muscle mass and strength in rodent models, in addition to its capacity to improve glucose homeostasis. Here, we tested the effects of a treatment with recombinant human FGF19 in a CKD mouse model, which associates sarcopenia and metabolic disorders. In 5/6 nephrectomized (5/6Nx) mice, subcutaneous FGF19 injection (0.1 mg/kg) during 18 days increased skeletal muscle fiber size independently of food intake and weight gain, associated with decreased gene expression of myostatin. Furthermore, FGF19 treatment attenuated glucose intolerance and reduced hepatic expression of gluconeogenic genes in uremic mice. Importantly, the treatment also decreased gene expression of liver inflammatory markers in CKD mice. Therefore, our results suggest that FGF19 may represent a novel interesting therapeutic strategy for a global improvement of sarcopenia and metabolic complications in CKD.

## Introduction

Chronic kidney disease (CKD) is a worldwide public health problem. CKD is widely considered as both a bone and muscle-weakening disease, eventually leading to frailty phenotype, with detrimental effects on overall morbidity^1–3^. Currently, there is no strategy for combating osteosarcopenia in CKD patients except physical activity which is strongly limited by compliance^4^.

We recently demonstrated that mice treated with human recombinant fibroblast growth factor-19 (FGF19) have larger skeletal muscles and increased muscle fiber surfaces^5^. Likewise, FGF19 increases the size of human myotubes in vitro^5^. At a signaling level, FGF19 binds to FGF receptors (FGFR)/ß-klotho complex and induces its hypertrophic effect by activating an extracellular-signal-regulated protein kinase 1/2 (ERK1/2)/ mammalian target of rapamycin (mTOR) pathway^5^. Importantly, FGF19 treatment during one or 2 weeks improves muscle wasting and muscle strength in different experimental models including sarcopenic aged mice and glucocorticoid-treated mice^5^. We further reported beneficial effect of one-week FGF19 treatment on muscle strength and locomotion parameters in a rat model of cerebral palsy^6^. Supporting these data, mice with specific intestinal Fgf15 knockout showed reduced muscle fiber size and altered bone metabolism^7^. Human FGF19, and its mouse ortholog Fgf15, are post‐prandial hormones produced by enterocytes from the ileum in the small intestine^8–10^. FGF15/19 is a master regulator of bile acid metabolism and an important player in lipid and carbohydrate metabolism^8,9^. Rodent studies showed that the elevation of circulating FGF15/19 levels by pharmaceutical treatment or by transgenic overexpression leads to improved metabolic parameters^8,11–14^.

FGF19, together with FGF21 and FGF23, belongs to the atypical endocrine family of FGFs^8^. In contrast to the canonical FGFs, endocrine FGFs act as circulating hormones and require either α- or β-klotho as co-receptors to activate their cognate FGFRs. While the metabolic roles of FGF23 and FGF21 have been explored in CKD, the function of FGF19 in the uremic context remains unclear^15^. A reduction of FGF19 secretion after a meal test was observed in CKD patients^16^, whereas another study reported a positive correlation between FGF19 and glucose parameters in CKD patients^17^.

Based on all these data, we hypothesized that FGF19 could be a potential new pharmacological target to counteract the loss of muscle, and to improve glucose homeostasis in CKD. Therefore, the aim of this study was to examine the potential benefits of human recombinant FGF19 treatment on musculoskeletal parameters and glucose metabolism in a mouse model of CKD which associates muscle wasting, renal osteodystrophy and perturbation of glucose homeostasis^18–21^.

## Results

### FGF19 protects against muscle wasting in CKD mice

**Figure 1:**
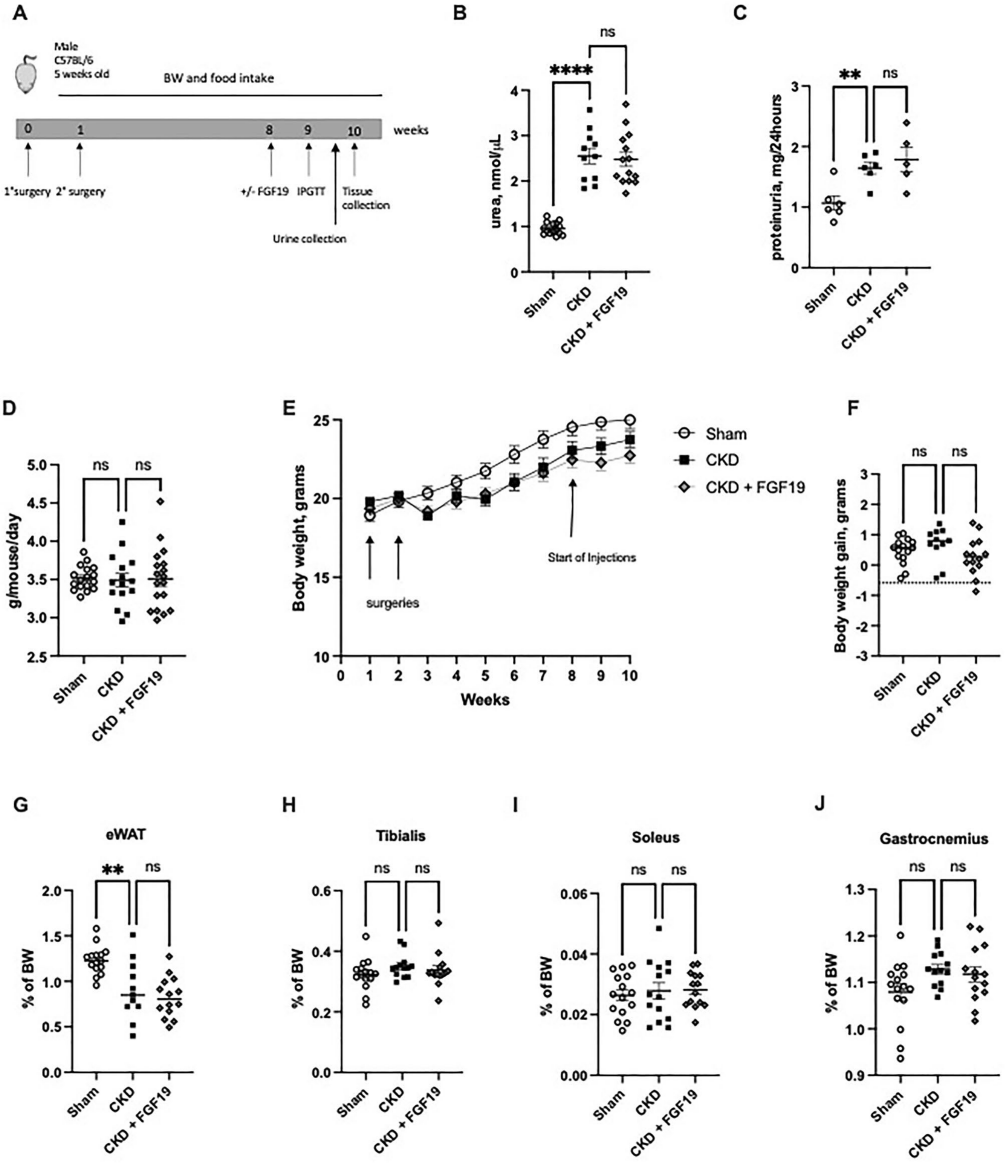
FGF19 treatment does not modify food intake and body composition in uremic mice. CKD mice induced by 5/6 nephrectomy or Sham mice were treated with 0.1 mg/kg body weight of FGF19 or with vehicle during 18 days. (**A**) Experimental timeline. (**B**) Blood urea. (**C**) 24 h proteinuria. (**D**) Daily food intake. (**E**) Body-weight evolution. (**F**) Body-weight gain. (**G**) Weight of epididymal WAT. (**H**) Weight of tibialis anterior, and (**I**) Weight of soleus (**J**) Weight of gastrocnemius after FGF19 or vehicle injection. n = 13–15 mice per group expected for proteinuria (n = 5–6). Data are mean ± SEM. Statistical analysis was done using a one-way ANOVA test. *P < 0.05, **P < 0.01, ****P < 0.0001. Abbreviation: BW: body weight, CKD: chronic kidney disease., n.s., not significant; WAT: white adipose tissue.

Six weeks after the 2nd surgery, Sham and CKD mice were treated during 18 days with FGF19 or its vehicle (Fig. 1A). In CKD + FGF19 mice, the mean plasmatic concentrations of FGF19 was 508 pg/mL. This corresponds to about twice the average concentrations found in CKD patients (Supplementary Figure 1)^15,18–20^. At sacrifice, circulating urea levels in CKD mice were significantly increased (*p* < 0.001), reaching values similar to those observed in moderate uremic patients (Fig. 1B)^22^. The 24 h proteinuria was also increased in CKD mice compared to Sham mice (*p* < 0.01) (Fig. 1C). These altered renal functions were not modified by FGF19 treatment (Fig. 1B, C). Daily food intake assessed between week 2 and 10 was similar in all groups (Fig. 1D). The body weight of Sham, CKD and CKD + FGF19 mice were similar before the two surgeries (Fig. 1E), whereas six weeks after the second nephrectomy, the body weights of CKD mice were significantly lower compared to Sham mice (*p* < 0.05) (Fig. 1E). In all three groups, mice gained similar body weight after the 2nd surgery (Fig. 1F). Compared with Sham mice, CKD mice displayed a significant decrease of epididymal adipose tissue (*p* < 0.01), and FGF19 treatment did not have any impact on uremic lipodystrophy (Fig. 1G).

**Figure 2:**
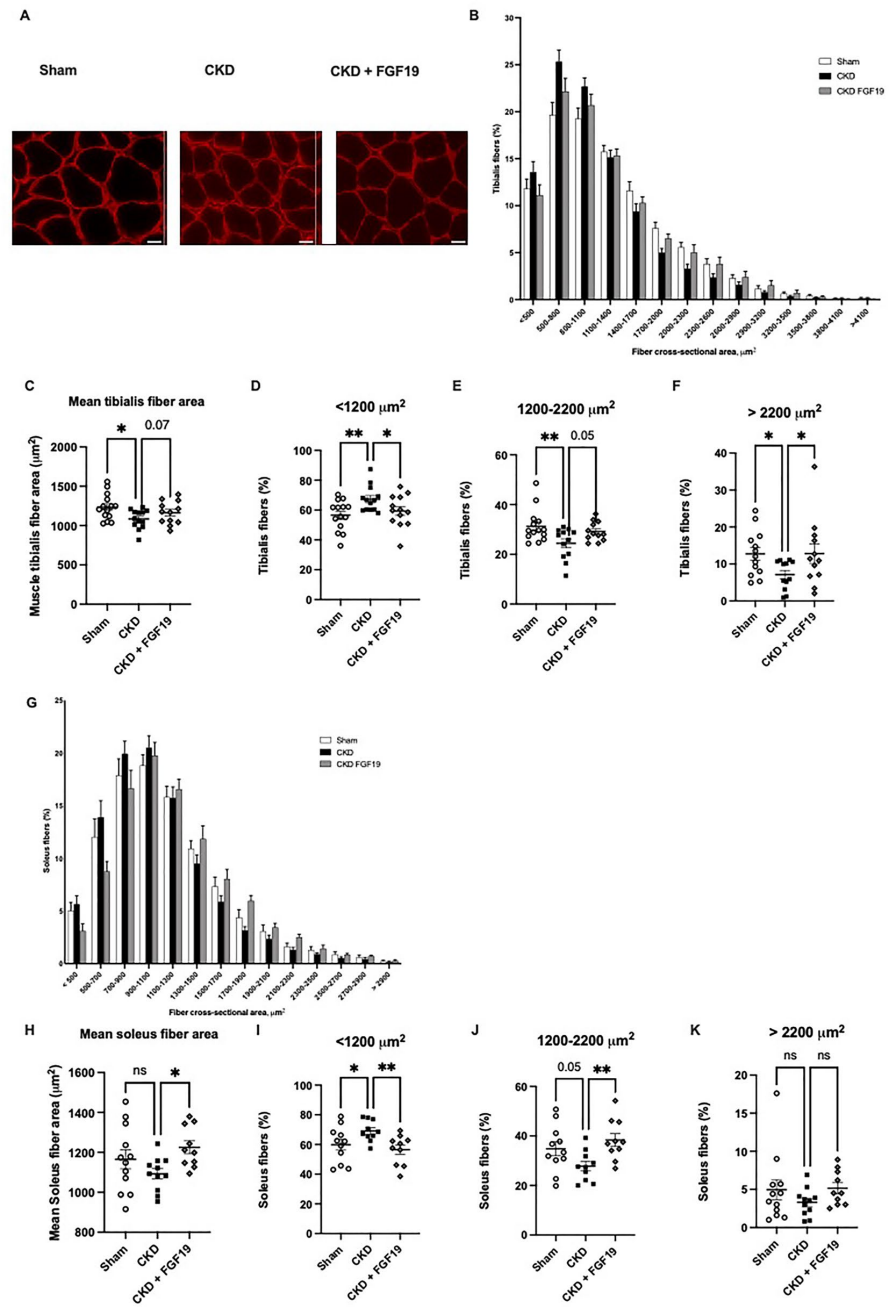
FGF19 treatment induces skeletal muscle hypertrophy in CKD mice. (**A**) Pictures are representative images of laminin-stained tibialis anterior muscles (n = 6–12 images per mouse), scale bar = 50 µm. (**B**) Frequency distribution of cross-sectional tibialis anterior fiber area. (**C**) Mean tibialis anterior fiber area. Distribution of fibers in tibialis anterior (**D**) < 1200 μm^2^, (**E**) between 1200 and 2200 μm^2^ and (**F**) > 2200 μm^2^. (**G**) Frequency distribution of cross-sectional soleus fiber area. (**H**) Mean soleus fiber area. Distribution of fiber in soleus (**I**) < 1200 μm^2^, (**J**) between 1200 and 2200 μm^2^ and (K) > 2200 μm^2^. Data are mean ± SEM. n = 11–15 mice per group. Statistical analysis was done using a one-way ANOVA test. *P < 0.05, **P < 0.01. Abbreviation: CKD: chronic kidney disease, n.s., not significant.

Before investigating the impact of FGF19 on skeletal muscle of CKD mice, we verified the impact of uremia and FGF19 treatment on the FGF15/19 signaling machinery in soleus muscle. No differences were found regarding the gene expression of the receptor *Fgfr4* and the co-receptor *Klb* (the mouse gene encoding β-Klotho) between the groups (Supplementary Figure 2). Then, because we previously demonstrated that FGF19 prevents muscle wasting by enlarging muscle fiber size and by protecting muscles from atrophy^5^, muscle mass and muscle cross section areas were analyzed in different skeletal muscles. There was no difference regarding tibialis anterior, soleus and gastrocnemius weight among groups (Fig. 1H–J). Histological analysis of tibialis anterior and soleus muscles revealed that although most muscle fibers showed normal morphology with regular and polygonal muscle fibers in Sham mice, the aspect of muscle fibers in CKD mice seemed more irregular (Fig. 2A). Examination of muscle-fiber size distribution in tibialis anterior further revealed that CKD mice had a shift toward small myofibers, as compared to Sham mice (Fig. 2B), resulting in a decrease of mean muscle tibialis anterior fiber area in uremic mice (1083 ± 33.58 vs 1235 ± 44.2 µm^2^ in Sham mice, *p* < 0.05) (Fig. 2C). As we previously observed in different models of muscle atrophy^5^, administration of FGF19 in CKD mice partially counteracted the decrease of muscle fiber surface induced by CKD (Fig. 2B). FGF19-treated CKD mice clearly presented a substantial rightward shift in the distribution of myofiber sizes (Fig. 2B, E–F), with a trend of increased cross‐sectional area average in tibialis anterior myofibers (1206 ± 57.91 vs 1083 ± 33.58 µm^2^ in CKD mice, *p* = 0.07) (Fig. 2C). Similarly, in the soleus muscle, the treatment with FGF19 also counteracted the CKD-induced defects in muscle fiber size, with a reduction of the number of small myofibers and an increase of myofibers within the range 1200–2200 µm^2^ (Fig. 2G–K). These results clearly showed that hypertrophic effect of FGF19 treatment is observed in two types of muscles (glycolytic tibialis anterior and oxidative soleus). In soleus, CKD mice were characterized by a higher expression of fast myosin genes (Myh4 (*p* < 0.05) and Myh1 (*p* = 0.11)) when compared to Sham animals, and FGF19 treatment tended to reduce this overexpression. However, when the fiber type was assessed by a myosin ATPase staining, we found that the proportion of type I and IIA/B fibers were not different between the groups (Supplementary Figure 1).

**Figure 3:**
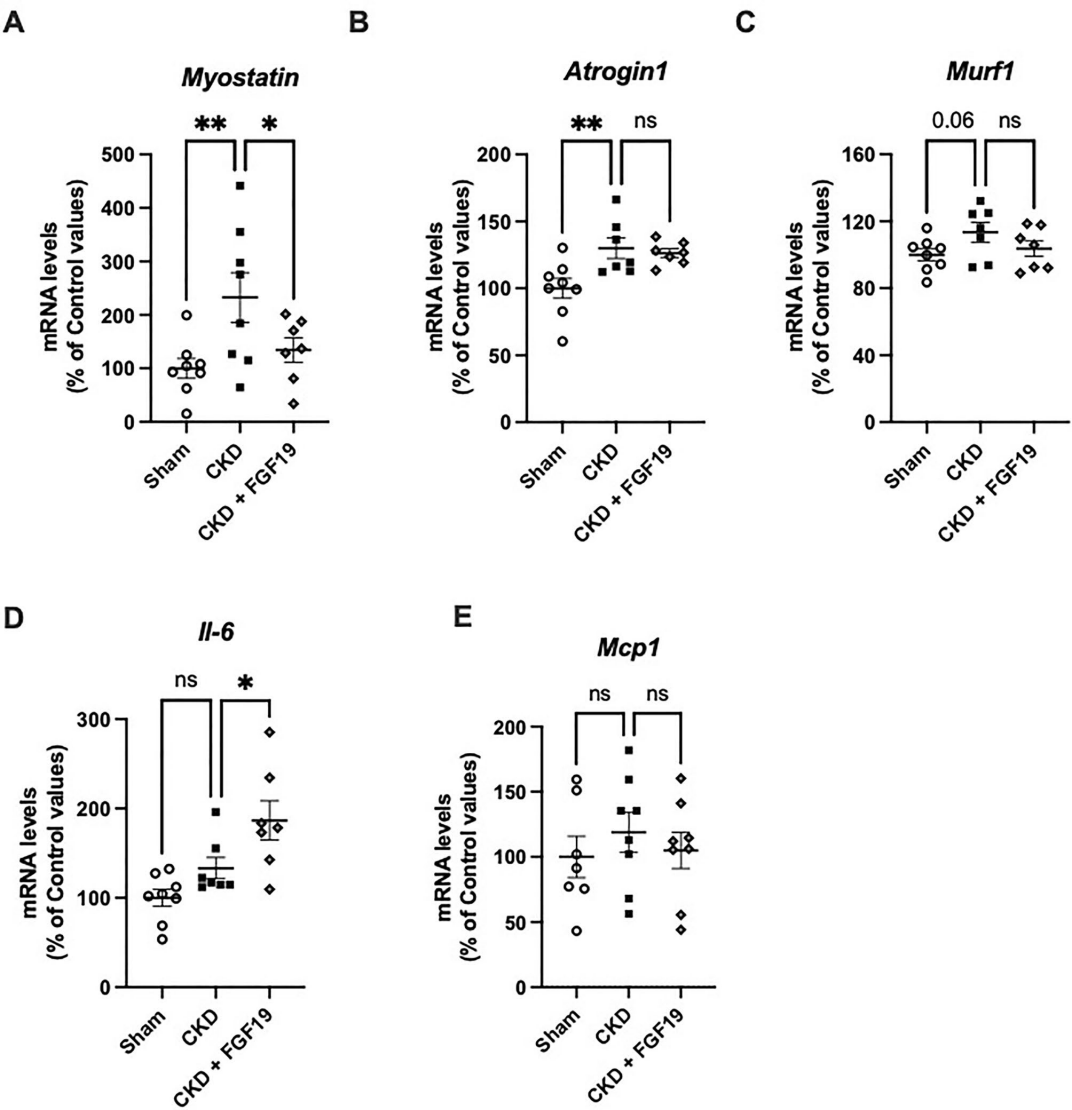
FGF19 partially decreases expression of negative regulators of muscle mass in soleus of CKD mice. Relative mRNA expression of (**A**) *Myostatin* (**B**) *Atrogin* (**C**) *Murf1* (**D**) *Il-6* and (**E**) *Mcp-1* in Sham and CKD mice after FGF19 or vehicle treatment. *Tbp* (TATA-Box Binding Protein) was used as reference gene to normalize the results. Results are expressed as the ratio of target mRNA levels to housekeeping gene mRNA levels and normalized to the levels in Sham mice. Data are expressed as mean ± SEM for n = 7–8 animals in each group. Statistical analysis was done using a one-way ANOVA test *p < 0.05, **p < 0.01. Abbreviation: CKD: chronic kidney disease., *Il-6*: interleukin-6, *Mcp1*: monocyte chemoattractant protein 1, n.s., not significant.

As skeletal muscle atrophy in CKD can result from several interrelated mechanisms leading to an imbalance between protein synthesis and degradation^4,23^, we further investigated whether the beneficial effects of FGF19 could implicate pathways such as the ubiquitin–proteasome or myostatin. Atrophy-related ubiquitin ligases *Atrogin-1* (+29%, p < 0.01), and *Murf1* (+14%, *p* = 0.06) were upregulated in soleus muscles from *CKD* mice as well as *Myostatin* (+132%, *p* < 0.01) (Fig. 3A–C). Treatment with FGF19 decreased significantly the expression of *Myostatin* (*p* < 0.05) (Fig. 3A) and reduced, albeit not significantly, *Murf1* (*p* = 0.17) (Fig. 3C). The treatment had no impact on *Atrogin-1* expression (Fig. 3B). Because it was proposed that the cytokine interleukin-6 (IL-6) in skeletal muscles contributes to energy metabolism and to the regulation of muscle mass^24^, we also measured the changes in *Il-6* mRNA abundance in muscles. There was no difference in *Il-6* expression in soleus muscle between Sham and CKD mice, but FGF19 treatment significantly increased *Il-6* mRNA levels (Fig. 3D). The gene expression of other inflammatory markers in soleus, like *Mcp1*, was not different between the groups (Fig. 3E). Given that reactive oxygen species (ROS) can play an important role in skeletal muscle atrophy and that some data suggest that FGF19 can attenuate oxidative stress^12^, we also measured the expression of key enzymes of oxidative stress defense in soleus. Results did not reveal any differences between groups (Supplementary Figure 4).

Bone loss is often correlated with muscle wasting in CKD^20,25,26^. We postulated that FGF19 treatment could eventually improve bone quality in parallel to its beneficial action on muscle parameters. As expected, the experimental CKD model induced a loss of bone, independently of body weight and diet (Supplementary Figure 5). CKD was associated with an increase in cortical porosity, a decrease in cortical thickness and a lower mineralization (tissue mineral density) without modification of the trabecular structure (Supplementary Figure 5B–H). However, the 18-days FGF19 treatment was not associated with any detectable effect on these bone structural parameters (Supplementary Figure 5A–H).

### FGF19 improves whole-body glucose tolerance and reduces hepatic expression of gluconeogenesis-related genes

**Figure 4:**
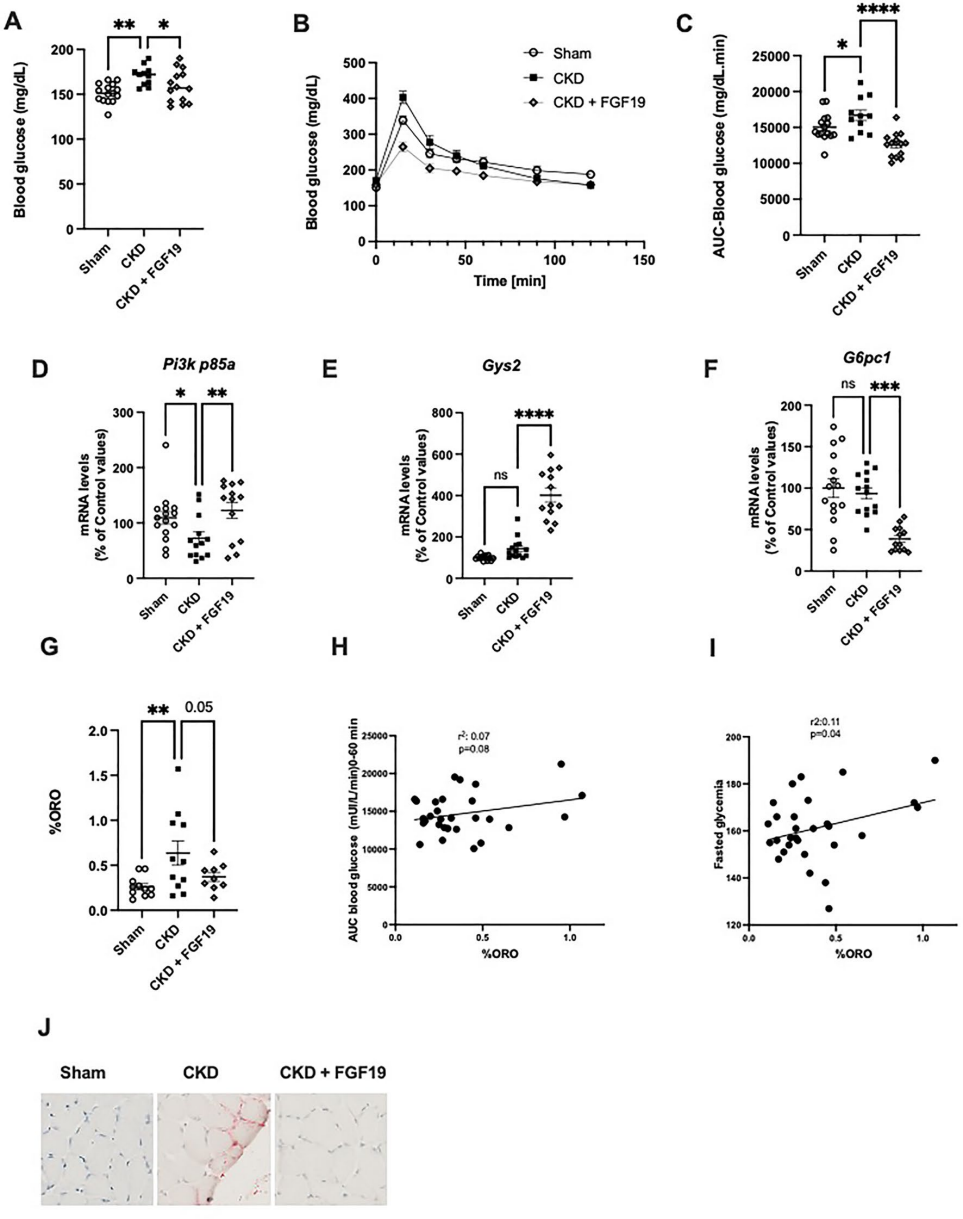
FGF19 corrects uremia-mediated insulin resistance in association with suppressed hepatic gluconeogenesis genes expression. (**A**) Fasted blood glucose. (**B**) Glucose levels during an i.p. GTT (2 g/kg) and (**C**) AUC (0–60 min) in control and CKD mice after FGF19 or vehicle treatment. Relative mRNA expression of (**D**) *Pi3K p85a* (**E**) *Gys2* and (**F**) *G6pc1* in Sham and CKD mice after FGF19 or vehicle treatment. (**G**) Percentage of Oil Red O staining of soleus muscle in Sham and CKD mice after FGF19 or vehicle treatment. Relationship between Oil Red O staining of soleus muscle and (**H**) fasted glycemia or (**I**) AUC glucose during ip-GTT. (**J**) Representative images of muscle sections stained with Oil Red O. *Tbp* (TATA-Box Binding Protein) was used as reference gene to normalize the results. Results are expressed as the ratio of target mRNA levels to housekeeping gene mRNA levels and normalized to the levels in Sham mice. Data are expressed as mean ± SEM for n = 9–15 animals in each group. Statistical analysis was done using a one-way ANOVA test *p < 0.05, **p < 0.01, ***p < 0.001. Abbreviations: AUC: area under curve, CKD: chronic kidney disease, *G6pc1*: glucose-6-phosphatase, *Gys2*: glycogen synthase, *Pi3k*: phosphoinositide 3-kinase, n.s., not significant.

CKD mice had higher fasting plasma glucose levels (170.7 ± 3.3 mg/dl in CKD mice vs 151.3 ± 7.7 mg/dl in Sham mice, *p* < 0.01) (Fig. 4A), and were glucose intolerant, as assessed by ip-GTT (Fig. 4B). After treatment with FGF19, the CKD mice displayed a marked reduction in their glucose excursion during ip-GTT (area under curve (AUC) 12,573 ± 456 vs. 16,693 ± 2465 mg/dl min^−1^, *p* < 0.0001, in FGF19-treated CKD mice vs CKD mice) (Fig. 4B, C).

The metabolic effects of FGF19 in the liver have been previously linked to a stimulation of hepatic glycogen metabolism and a reduction of gluconeogenesis through an insulin-independent pathway^9^. We found that treatment with FGF19 increased the hepatic gene expression of the regulatory subunit p85 of the *Pi3k* gene (Fig. 4D) and of glycogen synthase 2 (*Gys2*) (Fig. 4E) and reduced the expression of glucose-6-phosphatase (*G6pc1*) (Fig. 4F).

In addition to changes in liver metabolism, we previously reported that ectopic lipid accumulation in muscle correlates with insulin resistance in CKD mice model^27^. Here we found using an Oil Red O staining that lipid droplet infiltration was increased in soleus muscle of CKD mice and that FGF19 treatment mitigated this accumulation of lipids (Fig. 4G and J). Furthermore, in agreement with previous findings^27^, the ectopic accumulation of fat in skeletal muscle was correlated with insulin resistance parameters (Fig. 4H, I).

### FGF19 attenuates hepatic inflammation in CKD mice

**Figure 5:**
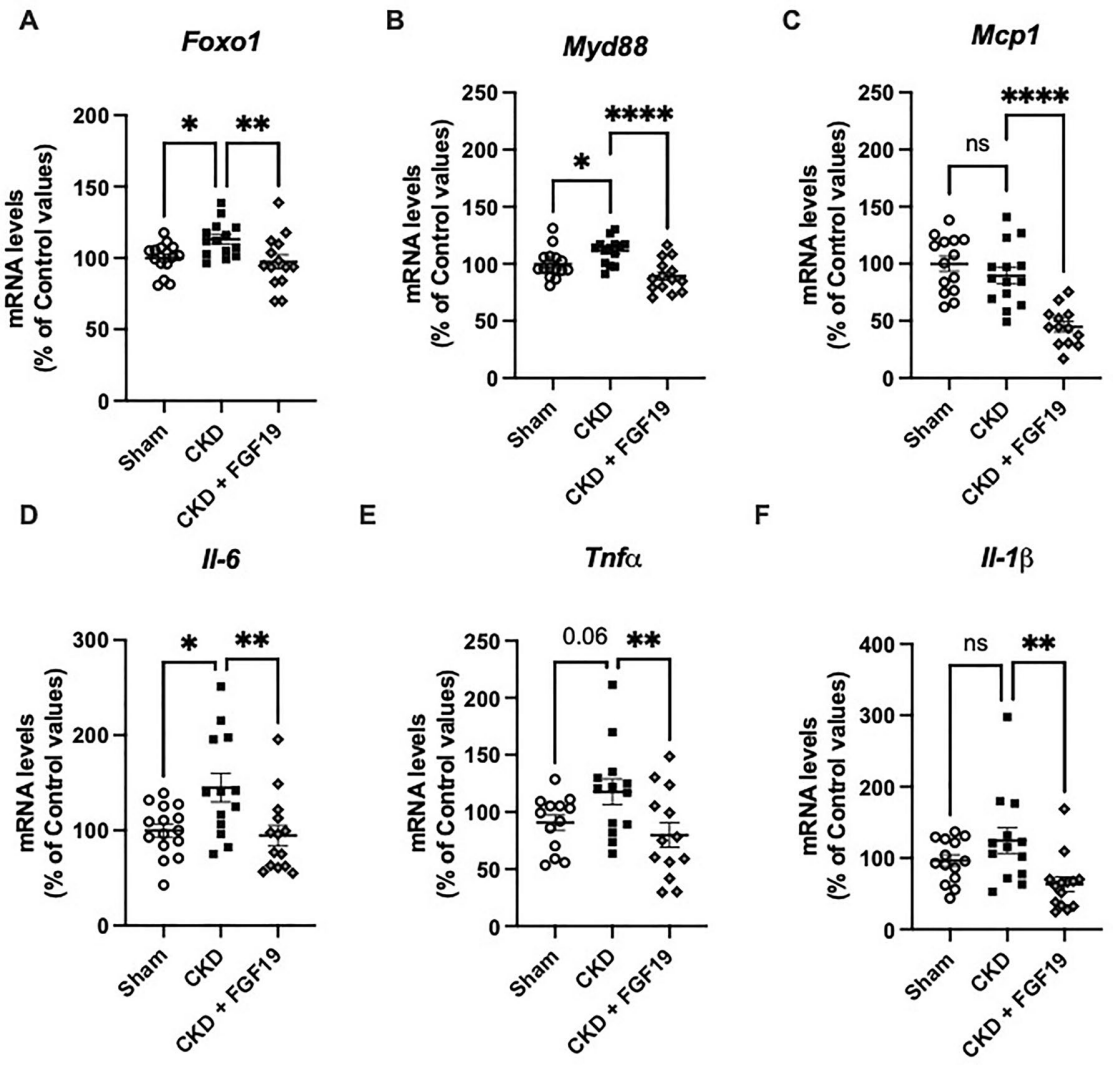
FGF19 reduces hepatic metabolic inflammation in CKD mice. Relative mRNA expression of (**A**) *Foxo1* (**B**) *Myd88* (**C**) *Mcp1* (D) *Il-6* (**E**) *Tnfα* and (**F**) *Il-1 β* in Sham and CKD mice after FGF19 or vehicle treatment. *Tbp* (TATA-Box Binding Protein) was used as reference gene to normalize the results. Results are expressed as the ratio of target mRNA levels to housekeeping gene mRNA levels and normalized to the levels in Sham. Data are expressed as mean ± SEM for n = 13–15 animals in each group. Statistical analysis was done using a one-way ANOVA test *p < 0.05, **p < 0.01, ***p < 0.001. Abbreviation: CKD: chronic kidney disease, *Foxo1*: Forkhead box protein O1, *Il:* interleukine *Mcp1*: monocyte chemoattractant protein 1, *Tnfα*: Tumor Necrosis Factor alpha, n.s., not significant.

Previous works have demonstrated a chronic state of inflammation in CKD models. Here, we found that CKD was associated with elevated expression of several inflammatory markers in the liver. Liver of CKD mice had increased gene expression of inflammatory cytokines (e.g. *Il-1β, Il-6, Tnfα),* of *Mcp1*, a chemokine that recruits and activates macrophage, of *Foxo1* a master regulator of inflammation and of *Myd88* a central adaptor of innate immunity. Interestingly, FGF19 treatment reduced significantly the expression levels of all these inflammatory markers (*Foxo1*, *Myd88*, *Mcp1*, *Il-6*, *Tnfα*, and *Il-1β*) in the livers of CKD mice (Fig. 5A–F). However, the levels of TNFα and IL-6 proteins measured by ELISA in liver extracts were very low and were not different between the groups (Supplementary Figure 6).

## Discussion

Muscle wasting is a well-established complication of CKD, which significantly worsens the quality of life of CKD patients^1–3^. This muscle deterioration is poorly understood, but likely involves multiple biological mechanisms^4^. Yet, no readily available treatment for successfully preventing muscle wasting in CKD exists. In this study, we tested the potential of FGF19 to protect skeletal muscle in 5/6 nephrectomized mice as a CKD model. Our results demonstrated for the first time that subcutaneous injections of human recombinant FGF19 during 18 days in uremic mice is able (1) to improve fiber size of skeletal muscles (2) reverse glucose intolerance and (3) improve the gene expression of inflammatory markers in the liver.

We have confirmed that FGF19 can be a promising agent to treat muscle wasting through increasing skeletal muscle fiber size^5,6^. In previous studies, we demonstrated that treatment with FGF19 during one or 2 weeks improved muscle wasting and muscle strength in different experimental models including sarcopenic aged mice and glucocorticoid-treated mice, but also found that FGF19 could have an anabolic effect in normal mice and in human myotubes^5^. In the present experiments, we further strengthened the therapeutic potential of FGF19 by demonstrating its ability to counteract CKD‐induced muscle atrophy. In our previous works^5^, we demonstrated that the hypertrophic effects of FGF19 on skeletal muscle fibers is mediated by the ERK/mTOR signaling pathway. Although we did not directly examine this pathway in the present work, we are expecting the same mechanism because the effect of FGF19 on muscle fiber size, without affecting fiber type composition, is rather similar to the one observed previously. However, one cannot exclude that other pathways might be also involved due to the uremic condition. This remains to be investigated specifically in further studies.

This beneficial effect of FGF19 treatment on muscle fiber surface was associated with the suppression of myostatin expression and interestingly with an overexpression of *Il-6* in soleus muscle. IL-6 is a pleiotropic cytokine, which is produced by muscle fibers as a myokine with critical action on muscle homeostasis, adapting glucose uptake to the energy requirements during exercise or stress^28^. It is important to note that we did not observed any changes in the gene expression of other inflammatory markers in the soleus muscle of CKD mice with or without FGF19. This suggested therefore that the overexpression of *Il-6* is not reflecting an inflammatory state in the muscle but rather a specific induction of the myokine. This finding suggests therefore a potential role of IL-6 in the effect of FGF19 on skeletal muscle homeostasis, which remains to be further investigated in different physiological conditions.

In the present CKD mouse model, the effect of uremic environment was mainly found on the size of the muscle fibers whereas muscle mass did not appear altered. Also, the repartition of fibers in CKD mice was very modestly modified. This differs from previous observations reporting decreased muscle mass, a decrease of type I and IIA fibers and an increase of type IIB fibers in the majority of CKD rodent models^19,29,30^. The difference may be due to the diet since we used a standard chow diet (17% proteins) while others^19,29,30^ fed mice with 40% proteins in order to increase uremic intoxication and catabolism. Also, our mice were younger and presented a less advanced CKD than in previous reports^31,32^. Nevertheless, the important observation here is the effects of FGF19 on muscle fiber surface and on the expression of genes related to muscle atrophy. In addition, it is important to note that FGF19 treatment was started 6 weeks after the second nephrectomy and not immediately after uremic induction, as it was generally done for other treatments, such as leptin receptor antagonist or myostatin inhibitors^19,33^. This may support FGF19 for curative treatment for uremic myopathy beside to be a preventive strategy.

The role of FGF19 on bone metabolism has remained poorly explored. A recent study suggested that Fgf15 is important to prevent bone loss after bariatric surgery in obese mice^7^. However, in the present CKD model, we failed to detect any effect of FGF19 treatment on bone parameters. The lack of effect of FGF19 while clear alterations were seen in the control CKD mice, may suggest that 18 days of treatment were not sufficient to impact bone parameters at a measurable level.

In agreement with previously published data using obese rodent models, we found that FGF19 did ameliorate glucose intolerance induced by CKD. Previous studies reported elevated hepatic glucose production in chronically uremic patients^34^ and also in CKD mice^35^. Inhibition of hepatic gluconeogenesis by FGF15/19 was previously observed in rodents^8,36^, and we found in the liver of CKD mice that FGF19 treatment reduced the expression of *G6pc1*, the rate limiting enzyme of gluconeogenesis. In addition, FGF19 stimulated the hepatic expression of *Gys2,* the main hepatic isoform of glycogen synthase. Together with the higher expression of p85subunit of *Pi3kinase* in the presence of FGF19, these data provided some mechanistic insights supporting the beneficial action of FGF19 on liver glucose metabolism and insulin action. Interestingly, studies have demonstrated that FGF19 levels is negatively correlated with fasting glucose and insulin levels in CKD patients^16,17^.

Previous data have shown that exogenous administration or genetic overexpression of FGF15 could decrease body weight and adiposity^11–13^ in healthy and pathological conditions. CKD mice have an increase of resting energy expense that participates to the development of sarcopenia^37^. In our study, we observed that treatment with FGF19 during 18 days did not change the final body weight and weight gain of CKD mice, suggesting that FGF19 does not affect energy expenditure in this model. Further studies are needed to explore more deeply this difference, which could be related to the conditions of the treatment or eventually to a specificity of the FGF19 action in the uremic context.

Hepatic inflammation is commonly observed in different CKD mice model^19^ and is associated with an increase in proinflammatory cytokines. Interestingly, we observed that, after 18 days of treatment with FGF19, the expression of several inflammatory markers in the liver of CKD mice was strongly inhibited. We found no difference in the amount of inflammatory proteins in the liver, but their levels were very low, limiting the possibility to highlight any potential statistical differences. Although the role of FGF19/15 on inflammation remains poorly documented, our findings are consistent with decreased hepatic inflammation and reversed liver injury in response to FGF19 treatment observed in a mouse model of primary sclerosing cholangitis^38^. Further studies are needed to investigate the potential anti-inflammatory action of FGF19.

All these preclinical data support a promising effect of FGF19 to fight metabolic alterations and skeletal muscle wasting in CKD. The effect of FGF19 itself has possibly CKD-independent effects on muscle and liver as previously published^5,8,36^. However, one cannot exclude additional actions of FGF19 on specific CKD defects. For example, uremic environment causes a lower density of growth hormone (GH) receptor and post-receptor defects in GH signal transduction, characterized by impaired phosphorylation and nuclear translocation of GH-activated STAT proteins^39^. This hormonal resistance could explain the failure of the positive effects of recombinant human growth hormone (hGH) in stable hemodialysis patients on lean body mass^40^. As a growth factor acting through an alternative signaling pathway compared to GH, FGF19 could eventually overcome this defect. Similarly, the effect of FGF19 on hepatic inflammation markers has not been evidenced so far^14^. We observed that the expression of genes related to inflammation are strongly down-regulated in CKD mice treated with FGF19. Further studies are thus needed to determine if FGF19 beneficial action in CKD mice could be related to different pathways than those previously described in a non-uremic context.

FGF15/19 acts by activating FGFR homodimers complexed with the membrane bound protein β-Klotho. CKD is characterized by a decrease of α-klotho gene and protein expression in kidney, and a lower level of circulating soluble α-klotho^41^. Also, CKD is associated with a decreases *Fgfr4* expression in kidney tissue^41^. However, the impact of CKD on the FGFR-β-Klotho pathway in muscles has never been explored. In this study, we did not see any difference on gene expression of *Fgfr4* and *Klb* in muscles of CKD mice and after FGF19 treatment. Several studies have reported an increase of FGF19 plasmatic levels in hemodialyzed patients^22,42^, suggesting that beyond renal accumulation, the high level of FGF19 in CKD could reflect a resistance of endogenous FGF19. We can state the hypothesis that the potential FGF19-resistance is not primarily due to a lowered *Fgfr4* and *Klb* expression in muscles but potentially by impaired signal transduction Therefore, our study is important to validate the proof of concept of the efficacy of FGF19 treatment in uremic context and in CKD patients.

In one clinical study, FGF19 secretion after a meal test was decreased in hemodialyzed patients^16^. FGF15/19 is produced by the ileal enterocytes and is released into the enterohepatic circulation postprandially in response to bile acids via activation of the farnesoid X receptor (FXR). Gut microbiota, as an indispensable participant in bile acids metabolism, is responsible for conversion of primary bile acids into secondary bile acids in the gut lumen and also for changes in the levels of several bile acid metabolites that could have different ability to activate FXR^43^. CKD is characterized by an intestinal dysbiosis^44^ and a modification of bile acids composition (decrease in the proportion of primary bile acids and increase in the proportion of secondary bile acids)^45^. Given these observations, it could be hypothesized that perturbations of bile acids metabolism linked to dysbiosis associated with CKD may have contributed to the reduced postprandial FGF19 response in this population^16^. Understanding the interplay between intestinal microbiota, bile acids metabolism and FGF19 secretion in CKD needs complementary studies.

This study has several limitations that deserve some comments. Firstly, as mentioned before, the CKD mice did not display a severe skeletal muscle atrophy and bone modifications. Previous studies highlighted an alteration of muscle function already measurable in young (12–15 weeks) 5/6 nephrectomized mice (like our model)^31,46^ despite that muscle volume and power are preserved, but generally, mineral and bone disorders are more intense in CKD mice 1 year after the nephrectomy^20,31^. Here we used juvenile mice of 6 weeks. It will be interesting to examine the long-term effects of FGF19 on skeletal muscle atrophy and potentially on bone parameters in an ageing-CKD model. Secondly, the study was performed in male mice only. Some studies have observed that the intensity of muscle wasting is less important in female mice in CKD models suggesting that the potential effect of FGF19 may be less significant in this population^47^. Also, Jia et al*.* reported that the expression of Fgf15 is higher in the skeletal muscle of female mice than that of male mice. Fasting reduces Fgf15 expression in female muscles but had no effect on male muscles^48^. The mechanisms underlying these gender differences are currently unknown and gender differences in FGF19 have never been explored and need further studies. Also, another important limitation of the present work is the fact that muscle function and muscle strength have been not measured. However, in all our previous studies, we have clearly demonstrated that the increase of muscle fiber size is associated with an increase of muscle strength^5,6^. Such association has been also found in CKD mice model^29,49^. Finally, a limitation for using FGF19 in human is the potential risk of hepatocellular carcinoma, as shown in transgenic mice overexpressing FGF19^50^. It has been observed that FGF19, as a growth factor, can stimulate tumor growth and invasion and promote several cancer types, including breast, prostate and colon cancer^51^. Interestingly, engineered FGF19 analogues devoid of pro-carcinogenic activity have been developed^36,52^ and one of them, Aldafermin (also known as NGM282 or M70), is currently in clinical development for the treatment of nonalcoholic steatohepatitis^36^. Aldafermin efficacy on skeletal muscle and potentially in CKD, remains to be evaluated.

In conclusion the presented preclinical data support a possible translational application of FGF19 therapy, or its analogues, in CKD patients. Without available treatment for CKD-associated sarcopenia validated to date, this novel strategy could provide a new way for the preservation of muscle mass in these patients.

## Materials and methods

### Animals

We confirm that all experimental protocols were approved by the local ethic committee named “comité d’éthique en expérimentation animale de la Région Rhône-Alpes, Lyon, France” (CECAPP # LS_2019_004). All experimental procedures were performed in accordance with the guidelines laid down by the French Ministry of Agriculture (no 2013–118) and the European Union Council Directive for the protection of animals used for scientific purposes of September 22nd, 2010 (2010/63UE). We confirm that all methods were carried out in accordance with relevant guidelines and regulations. We confirm that all methods are reported in accordance with ARRIVE guidelines (http://www.nc3rs.org.uk/arrive-guidelines). We confirm that during the surgery and the euthanasia, we have employed anesthesia consistent with the commonly accepted norms of veterinary best practice. All efforts were made to minimize the number of animals used and their suffering. The required sample size was calculated by a power analysis before the start of the experiment. Male C57BL/6j mice were group-housed in an air-conditioned room with a controlled environment of 21 ± 0.5 °C and 60–70% humidity with a 12 h light/dark cycle. Moderate CKD was induced by 5/6 nephrectomy with a 2-steps surgical procedure in 5-weeks-old male mice as previously described under anesthesia ketamine/xylazine (100 mg/kg and 20 mg/kg, intraperitoneal respectively)^18^. Sham mice underwent two mock surgeries. Body weight and food intake were measured once a week. Recombinant human FGF19 (R&D System, UK) was injected subcutaneously every day at 0.1 mg/kg during 18 consecutive days, 6 weeks after the second nephrectomy. The controls were injected with the vehicle solution, which consists of a phosphate buffer solution (PBS) with 0.1% of bovine serum albumin (BSA)). We had three groups of 15 mice: (1) Sham treated with vehicle solution; (2) CKD treated with vehicle solution and (3) CKD treated with FGF19. Two days before the end of the experiment, mice, by two, were placed in metabolic cages in order to collect the urine produced over 24 h. At the end of the study, mice were euthanized with anesthetic ketamine/xylazine (100 mg/kg and 20 mg/kg, intraperitoneal respectively).

### Glucose tolerance test, tissues and blood collection

For intraperitoneal glucose tolerance test (ip-GTT), animals were injected i.p. with 2 g of D-glucose/kg of body weight after a 5-h fast. D-glucose was diluted at 20% in PBS before the ip-GTT. Blood glucose was measured prior to and 15, 30, 45, 60, 90, and 120 min after the i.p. injection of D-glucose. Blood glucose values were determined from a drop of blood sampled from the tail using an automatic glucose monitor (Accu-Check® *Performa*, Roche*Diabetes*), as previously reported.^18^.

One week after the ip-GTT, mice were euthanized with ketamine/xylazine (70/4 mg/kg). Plasma was collected and stored at −80 °C. Skeletal muscles, liver and epididymal white adipose tissue were removed, and weighted. Only liver, soleus and tibialis muscles were snap-frozen in liquid nitrogen and then stored at −80 °C until analysis. A sample of soleus and tibialis anterior muscles from the controlateral leg were embedded in OCT-tissue freezing medium (General Data) for histological analysis. The mouse leg, without muscle tissue, was then stored in ethanol 80% at 4 °C until further bone-related analysis.

Plasma urea levels were measured using a commercial colorimetric assay kit (Sigma). Groups of two mice were placed in metabolic cages to collect urine during 24 h. Total protein levels in urine samples were determined using a standard Bradford protein assay (Bio-Rad Laboratories).

Plasma FGF19 concentrations were measured using a commercial colorimetric assay kit (R&D Systems).

### Gene expression analysis and inflammatory protein assays

Total RNAs from soleus muscles and livers were extracted using TRI Reagent® (Sigma Aldrich). Purity and concentration of RNA were determined using NanodropOne (Ozyme) and quality checked using Bioanalyser (Agilent). First-strand cDNAs were synthesized from 1 µg of total RNAs using PrimeScript RT kit reagent kit (Perfect Real Time) (Takara Bio Europe). Real-time qPCR assays were performed on 1/20 diluted cDNA using TB Green Premix Ex Taq (Tli RNaseH plus) (Takara Bio Europe) with Rotor-Gene 6000 (Qiagen) using TB Green Premix Ex Taq (Tli RNaseH plus) (Takara Bio Europe) as previously described^53^. TATA-box binding protein (*tbp*) was used as a reference gene to normalize the results. Results are the ratio of target mRNA levels to *tbp* mRNA levels and are expressed as percentage of the Sham group values. Primers sequences are listed in Supplementary Table 1. The protein levels of IL-6 (Abcam) and TNF-α (Abcam) were measured in the liver according to the manufacturer's recommendations.

### Muscle cross-sectional area

Fixed samples of tibialis anterior and soleus muscles were cryosectioned (10 μm-thick cryosections taken at the mid-belly of the muscles) and processed for immunostaining, as described previously^5^. Briefly, sections were blocked for 1 h at room temperature and incubated overnight at 4˚C with a rabbit anti-laminin antibody (Sigma, L9393), followed by incubation with a secondary antibody (AlexFluor Goat anti Rabbit IgG Alexa Fluor 594 -A11012 ThermoFisher). The 10× magnification images were taken using a BX63 Olympus upright microscope. The Fiji software was configured to take into account only the transverse fibers with a Ferret ratio strictly up to 0.5. The muscle fiber area was measured in μm^2^.

Oil Red O stain was used to determine the presence of lipid accumulation in the muscle tissue. Cryosections were rinsed in 60% isopropanol before staining with Oil Red O stain solution. Stain solution is prepared from a stock solution (0.5 g Oil Red O in 100 ml isopropanol) dissolved 3:2 in dH2O. Slides were incubated for 15 min in stain solution, before rinsing with 60% isopropanol.

The muscle typology was determined by the staining of the ATPase myosin. Briefly, 10 μm frozen muscle sections were pre-incubated in a calcium chloride solution (pH 4.2) for 4 min, washed twice for 30 s with Tris buffer (pH 7.8) and incubated for 25 min at 37 °C in a calcium chloride solution (pH 9.4) containing glycine buffer and 2.75 mM ATP disodium salt (Sigma-Aldrich, A2383). The sections were immersed 4 times for 30 s in a calcium chloride solution and incubated in a cobalt chloride solution for 3 min. Ammonium sulphide at 1% (v/v) (Santa Cruz Biotechnology, sc-214536) was used to precipitate cobalt into a new incubation bath. The frozen sections were then rinsed with water for 5 min and incubated in deionized water. The sections were soaked in 70/90/100% ethanol and mounted with Canadian balm mounting medium. At the end of the procedure, the Type I fibers were dark brown while the Type II fibers were colorless. Both types were counted manually and a percentage was calculated on each muscle area.

### Bone microarchitecture and cortical porosity

Microarchitecture was assessed for the cortical and trabecular bone of the femur (diaphysis and distal metaphysis, respectively), using high-resolution micro-Computed Tomography (VivaCT40, Scanco Medical) at 10.5 μm^3^ isotropic cubic resolution^54^. Data were acquired at 70 keV, with a 114 mA current and integration time of 275 ms. Three-dimensional reconstructions were generated using the following parameters: σ = 1.2; support = 2; threshold = 120 for trabecular bone and 0.8, 1, 260 for cortical bone.

The following structural parameters of cortical (Ct.) bone were measured from a stack of 60 sections: cortical porosity (Ct.porosity = (1 – (bone volume / total volume (BV/TV), %)), cortical thickness (Ct.Th, mm), cortical tissue mineral density (TMD, mg of hydroxyapatite (HA)/cm^3^). The following parameters of trabecular (Tb.) bone were measured from a stack of 150 sections: trabecular spacing (Tb.Sp), trabecular number (Tb.N., mm^−1^) and trabecular thickness (Tb.Th, mm), trabecular bone volume / total volume (Tb.BV/TV, %).

### Statistical analysis

GraphPad Prism 8 (GraphPad Inc.) was used to analyze the data. All quantitative data are expressed as the mean ± standard error of mean (SEM). Distributions were tested for normality using the D’Agostino-Pearson test. All individual points are shown. All variables were analyzed using one-way ANOVA as the independent variables. *P* value < 0.05 was considered to indicate statistically significant differences.

## Supplementary Information

Supplementary Information.

## Data Availability

The dataset is available on reasonable request from the corresponding author.
