# Supplementary material for: Treatment with fibroblast growth factor 19 increases skeletal muscle fiber size, ameliorates metabolic perturbations and hepatic inflammation in 5/6 nephrectomized mice
Source: Sci Rep. 2023 Apr 4;13:5520. doi: 10.1038/s41598-023-31874-4 (PMC10073190; doi:10.1038/s41598-023-31874-4)

Supplementary Table 1 : Sequence of primers used for qPCR analysis

Gene symbol	Official gene name	Forward primer sequence (5' - 3')	Reverse primer sequence (5' - 3')
Atrogin1	F-Box Protein 32	CTCTGCCAGTACCACTTCTC	ATGGTCAGTGCCCTTCCAGG
Cat	Catalase	TCAGGATGTGGTTTTCACTG	GTGTAAAATTTCACTGCAAAC
Fgfr4	Fibroblast growth factor receptor 4	CAACTCCATCGGCCTTTCCT	CAGAACCAGTGAGCCTGATA
Foxo1	Forkhead box protein O1	AGATCTACGAGTGGATGGTG	GGACAGATTGTGGCGAATTG
G6pc1	Glucose-6-phosphatase	TTACCAAGACTCCCAGGACTG	GAGCTGTTGCTGTAGTAGTC
Gpx1	Glutathione peroxidase 1	GTGAGCCTGGGCTCCCTGCG	ACTTGAGGGAATTCAGAATC
Gpx4	Glutathione peroxidase 4	CCGGCTACAACGTCAAGTTT	CACGCAGCCGTTCTTATCAA
Gys2	Glycogen synthase 2	TGTTTCGTGCAGGAGCATGTG	TTTGCCGGCGTGACTGTTTAC
Il-1β	Interleukin 1 beta	ACTGTTCCTGAACTCAACTG	CTTGTTGATGTGCTGCTGCG
Il-6	Interleukine 6	AGTTGCCTTCTTGGGACTGAT	TCCACGATTTCCCAGAGAAC
Klb	Klotho beta	AGCCAATGGCATCGATGAC	ACACGCAGGACTTCTGTTCT
Mcp1	Chemokine (C-C motif) ligand 2	TGGAGCATCCACGTGTTGGC	ACTACAGCTTCTTTGGGACA
Mstn	Myostatin	TGCTGTAACCTTCCCAGGACC	GTGCTCATCGCAGTCAAGCCC
Murf1	Tripartite motif-containing 63	TGCATCTCCATGCTGGTGGC	CTTCTTCTCGTCCAGGATGG
Myd88	Myeloid differentiation primary response 88	CTAGAACAGACAGACTATCGG	TGCAAGGGTTGGTATAGTCG
Myh1	Myosin heavy polypeptide 1	TGAGTGAGCTGAAGACCAAG	GAGAGCTGGGAAACTAATGA
Myh2	Myosin heavy polypeptide 2	AGGCGGCTGAGGAGCACGTA	GCGGCACAAGCAGCGTTGG
Myh4	Myosin heavy polypeptide 4	CGAGCAAGAGCTACTGGATG	TTGGCCTTCTCTTCTGCGTT
Myh7	Myosin heavy polypeptide 7	GCATCAAGGAGCTCACCTAC	GTAGCACAAGAGCTACTCCT
Pi3k p85a	Phosphoinositide-3-kinase, regulatory subunit 1 (p85 alpha)	CCGTTGAAATGCATAACCTGC	GTTTTTCATTCACTTCTTCCC
Sod1	Superoxide dismutase 1	TGAAGAGAGGCATGTTGGAG	CCACCTTTGCCCAAGTCATC
Sod2	Superoxide dismutase 2	TCATGCAGCTGCACCACAGC	CCATTGAACTTCAGTGCAGG
Tnfα	Tumor necrosis factor alpha	CCAGACCCTCACACTCAGATC	CACTTGGTGGTTTGCTACGAC

Supplementary Figure 1

Plasmatic FGF19 concentration.

Data are expressed as mean ± SEM for n =3-13 animals in each group. Statistical analysis was done using a one-way ANOVA test. **P < 0.01. Abbreviation: CKD: chronic kidney disease.

Supplementary Figure 2

FGF19 and CKD did not modify the expression of β-Klotho and fibroblast growth factor receptor 4 (FGFR4) in muscle.

Relative mRNA expression of (A) *Fgfr4* in soleus, (B) *β-Klotho* in soleus from Sham and CKD mice after FGF19 or vehicle treatment. *Tbp* (TATA-Box Binding Protein) was used as reference gene to normalize the results. Results are expressed as the ratio of target mRNA levels to housekeeping gene mRNA levels and normalized to the levels in Sham. Data are expressed as mean ± SEM for n =7-8 animals in each group. Statistical analysis was done using a one-way ANOVA test. Abbreviation: CKD: chronic kidney disease, *Fgfr4:* fibroblast growth factor receptor 4.

Supplementary Figure 3

FGF19 have no impact the repartition of muscle fiber in CKD mice.

Relative mRNA expression of (A) *Myh7*, (B) *Myh1*, (C) *Myh2*, and (D) *Myh4* in soleus from Sham and CKD mice after FGF19 or vehicle treatment. *Tbp* (TATA-Box Binding Protein) was used as reference gene to normalize the results. Myh7 is represented as a slow muscle type (type I), whereas Myh2, Myh1, and Myh4 are fast muscle types and are subclassified as type IIa, IIx, and IIb respectively. Results are expressed as the ratio of target mRNA levels to housekeeping gene mRNA levels and normalized to the levels in Sham. Data are expressed as mean ± SEM for n =7-8 mice per group. (E) Quantification of Type I and Type IIA/B fibers in soleus using an ATPase staining. Data are expressed as mean ± SEM for n =8-10 mice per group. Statistical analysis was done using a one-way ANOVA test. * P < 0.05. Abbreviation: CKD: chronic kidney disease, Myh: myosin heavy chain.

Supplementary Figure 4

Skeletal muscle mRNA expression in soleus muscle of oxidative stress defense markers.

Relative mRNA expression of (A) *Gpx1,* (B) *Gpx2,* (C) *Sod1,* (D) *Sod2*, and (E) *Catalase* in soleus from Sham and CKD mice after FGF19 or vehicle treatment. *Tbp* (TATA-Box Binding Protein) was used as reference gene to normalize the results. Results are expressed as the ratio of target mRNA

levels to housekeeping gene mRNA levels and normalized to the levels in Sham. Data are expressed as mean ± SEM for n =7-8 animals in each group. Statistical analysis was done using a one-way ANOVA test. Abbreviation: CKD: chronic kidney disease, *Gpx*: glucathion peroxydase, *Sod:* superoxide dismutase.

Supplementary Figure 5

FGF19 does not improved bone quality and porosity in CKD mice.

(A) Femur length in Sham and CKD mice after FGF19 or vehicle treatment. Bone structure was assessed by micro-Computed Tomography for (B) Cortical porosity (Ct. Po), (C) Cortical thickness (Ct.Th), (D) Cortical tissue mineral density (Ct. TMB), (E) Trabecular Bone Volume/Total Volume (Tb. BV/TV), (F) Trabecular thickness (Tb. Th), (G) Trabecular number (Tb.N.) and (H) Trabecular spacing (Tb. Sp). Data are mean ± SEM for n =13-15 animals in each group. Statistical analysis was done using a one-way ANOVA test. $^{**}P < 0.01$ and $^{***}P < 0.001$. Abbreviation: CKD: chronic kidney disease.

Supplementary Figure 6

FGF19 did not modify the amount of inflammatory proteins in liver in CKD mice.

Quantity of (A) TNF-α and (B) IL-6 in liver. Data are expressed as mean ± SEM for n =6-8 animals in each group. Statistical analysis was done using a one-way ANOVA test. Abbreviation: CKD: chronic kidney disease, Il: interleukine, Tnfα: tumor necrosis factor alpha.

Supplementary Figure 1

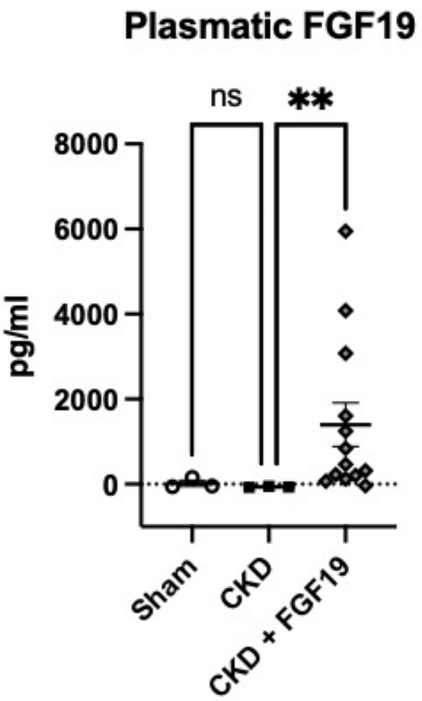

Supplementary Figure 2

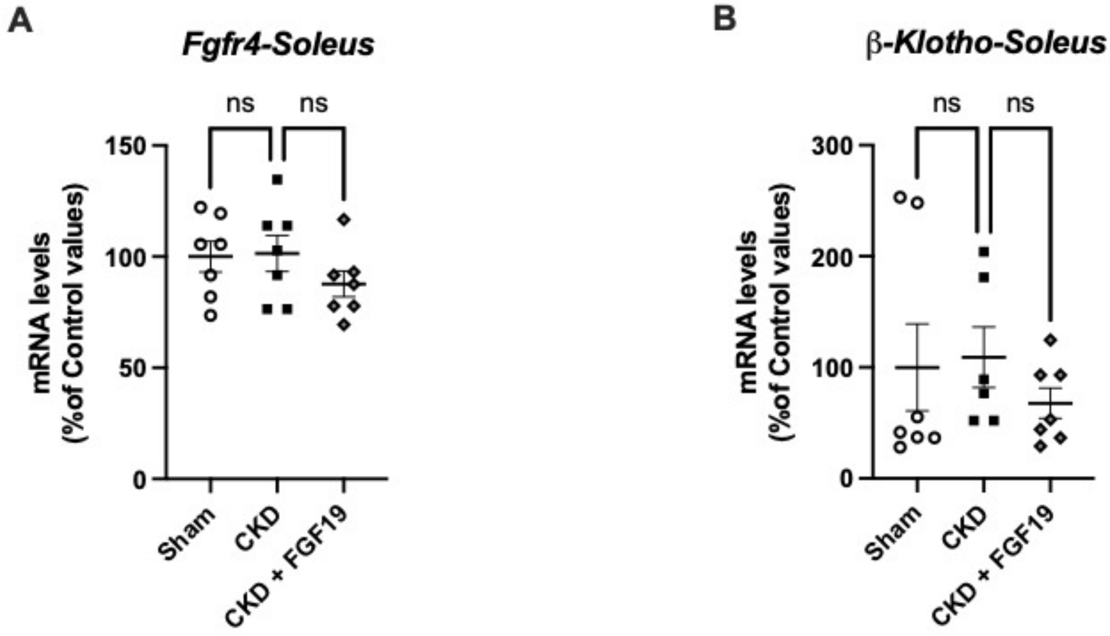

Supplementary Figure 3

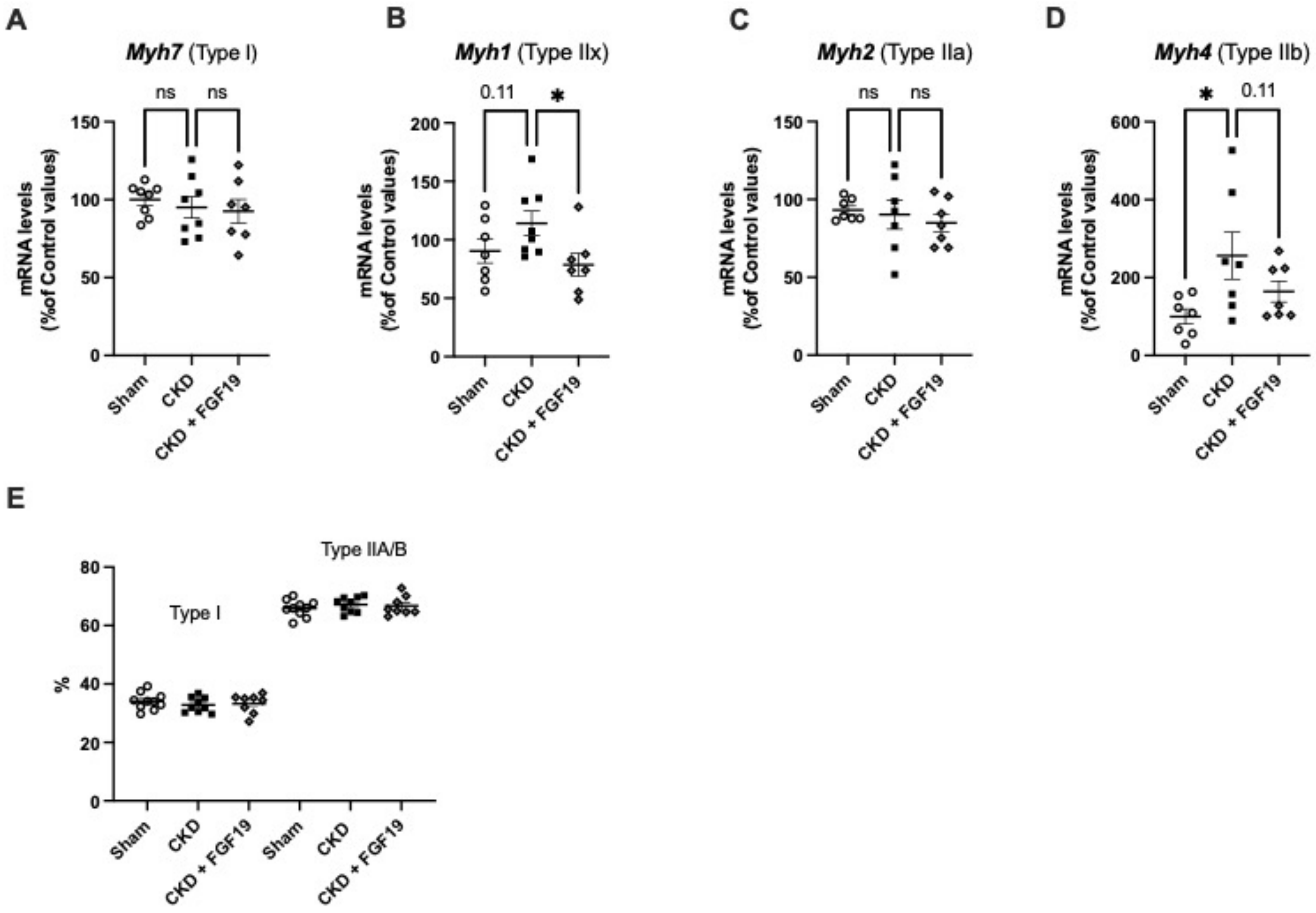

Supplementary Figure 4

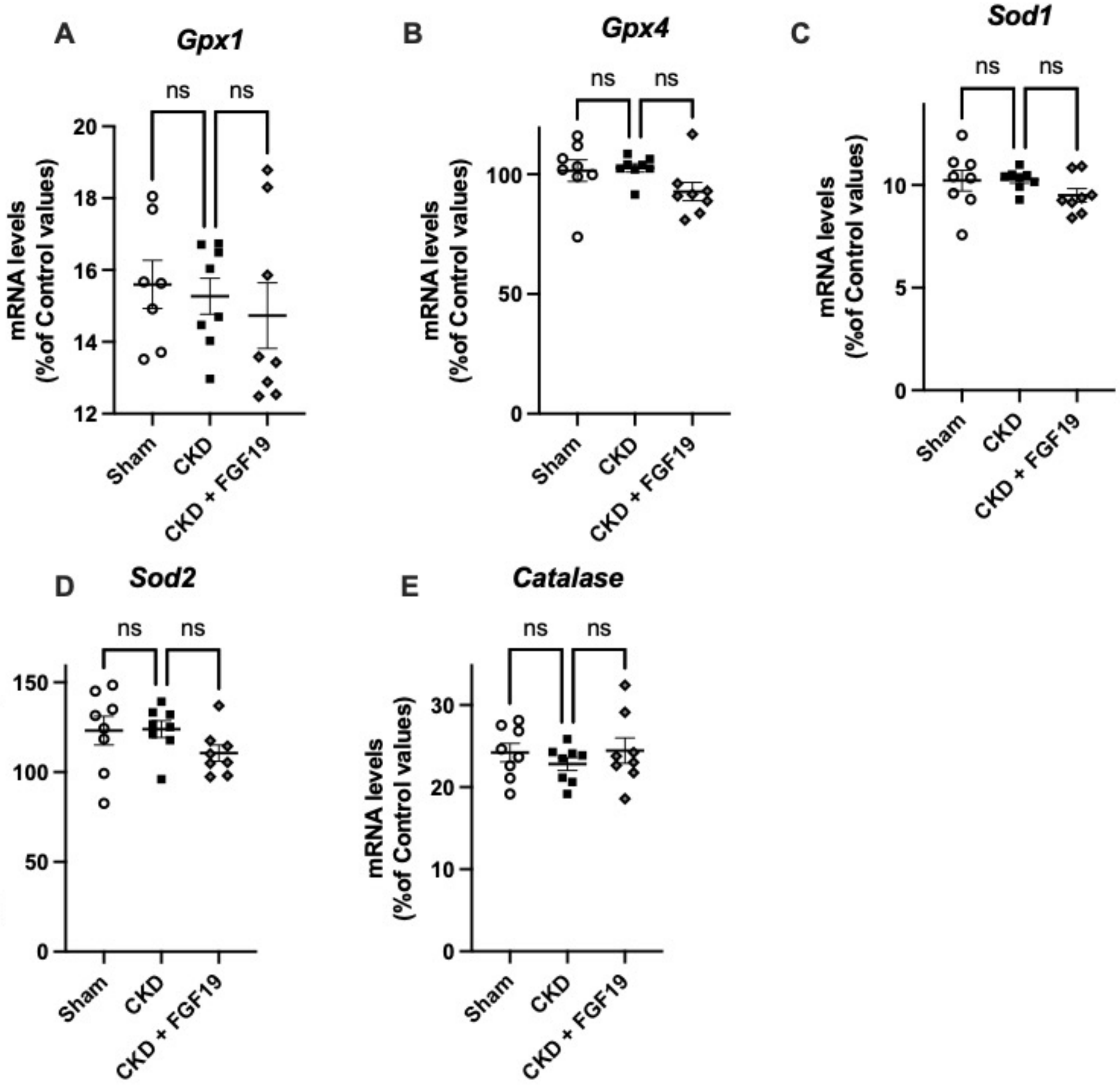

Supplementary Figure 5

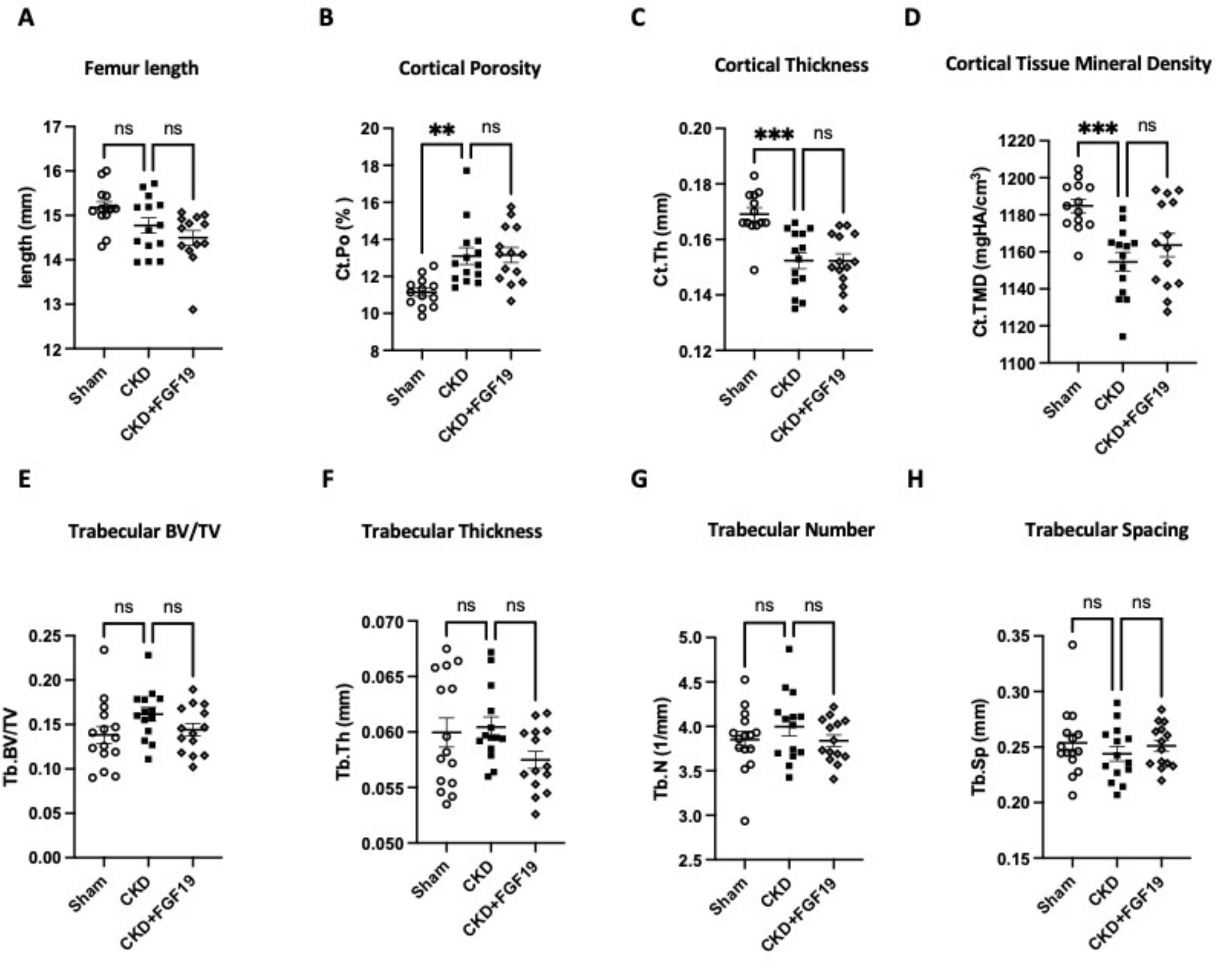

Supplementary Figure 6

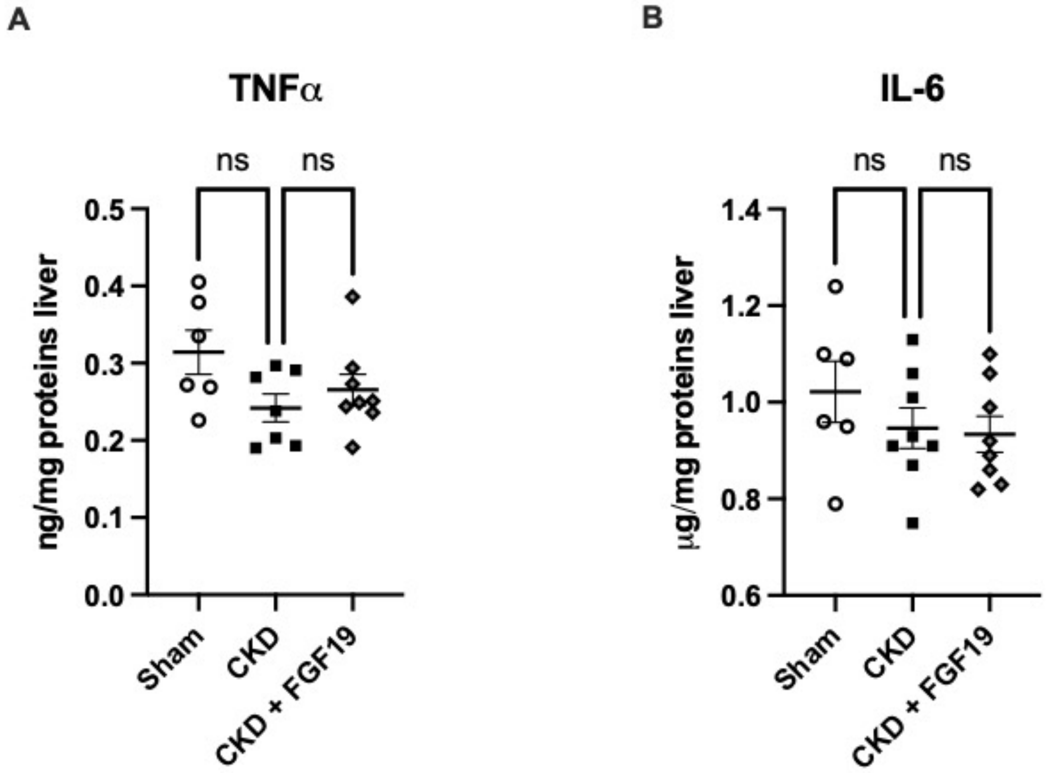

Supplement: Supplementary file 1 — Supplementary Information. [file 41598_2023_31874_MOESM1_ESM.pdf]
